# Supplementary material for: Selective Formation of Unsymmetric Multidentate Azine-Based Ligands in Nickel(II) Complexes
Source: Molecules. 2022 Oct 11;27(20):6788. doi: 10.3390/molecules27206788 (PMC9608849; doi:10.3390/molecules27206788)
Supplement: Supplementary file 1 [file molecules-27-06788-s001.zip › molecules-1972784-supplementary.pdf]

Supporting Information

Selective Formation of Unsymmetric Multidentate Azine-Based Ligands in Nickel(II) Complexes

Kennedy Mawunya Hayibor,[1] Yukinari Sunatsuki,[2] Takayoshi Suzuki [1,3]*

[1]Graduate School of Natural Science and Technology, Okayama University, Okayama 700-8530, Japan

[2]Advanced Science Research Center, Okayama University, Okayama 700-8530, Japan

[3]Research Institute for Interdisciplinary Science, Okayama University, Okayama 700-8530, Japan

Syntheses of the symmetrical and unsymmetrical azine compounds

Pyridine-2-carboxaldehyde azine (PAA)

A methanol solution (30 mL) of pyridine-2-carboxaldehyde (5.36 g, 50.0 mmol) was added to a methanol solution (30 mL) of hydrazine monohydrate (1.26 g, 25.0 mmol), and the mixture was refluxed with stirring at *ca* 60 °C for 5 h. The resulting precipitate was collected by filtration, and the filtrate was concentrated under a vacuum to obtain an extra precipitate. The combined precipitate was sequentially washed several times with acetonitrile, ethanol, and methanol, and dried in a vacuum. Yield: 5.24 g (99.7%). ^{1}H NMR (DMSO-d_6), δ: 8.00 (ddd, J = 4.8, 1.8 and 1.0 Hz, 1H), 7.84 (s, –CH=N, 1H), 7.40 (dt, J = 7.9 and 1.1 Hz, 1H), 7.26–7.21 (m, 1H), 6.81 (ddd, J = 7.6, 4.8 and 1.2 Hz, 1H): FTIR (KBr pellets; cm^{-1}): 1630 (C=N diimine, stretching), 1580 (C=N pyridine ring) and 1460 (C=C stretching).

Imidazole-4-carboxaldehyde azine (H_2ICA^H)

A methanol solution (30 mL) of 1*H*-imidazole-4-carbaldehyde (0.961 g, 10.0 mmol) was added to a methanol solution (30 mL) of hydrazine monohydrate (0.258 g, 5.00 mmol). The mixture was refluxed with stirring for 5 h and concentrated under reduced pressure, giving an amorphous pale-yellow residue. The residue was washed with methanol and ethanol several

times and dried over P_2O_5 in a desiccator. Yield: 0.922 g (98.0%). ^{1}H NMR (DMSO-d_6), δ: 12.68 (d, J = 14.3 Hz, 1H), 8.53–8.40 (m, –CH=N, 1H), 7.75 (dd, J = 33.2 and 10.5 Hz, 1H), 7.46 (d, J = 14.3 Hz, 1H): FTIR (KBr pellets; cm^{-1}): 3200–2600 (N–H, stretching), 1635 (C=N diimine, stretching), 1540 (C=N imidazole ring) and 1380 (C=C stretching).

2-Methylimidazole-4-carboxaldehyde azine (H_2ICA^{Me})

A methanol solution (30 mL) of 2-methyl-1*H*-imidazole-4-carbaldehyde (0.159 g, 1.44 mmol) was transferred to a methanol solution (30 mL) of hydrazine monohydrate (0.038 g, 0.72 mmol). The mixture was refluxed with stirring for 5 h and concentrated under reduced pressure, giving a yellowish powder. The powder was washed with methanol and ethanol several times and dried over P_2O_5 in a desiccator. Yield: 0.154 g (98.9%). ^{1}H NMR (DMSO-d_6), δ: 12.34 (d, J = 19.8 Hz, 1H), 8.39 (m, –CH=N, 1H), 7.71–7.17 (m, 1H), 3.37 (s, 1H): FTIR (KBr pellets; cm^{-1}): 3400–2800 (N–H, stretching), 1632 (C=N diimine, stretching), 1580 (C=N imidazole ring) and 1420 (C=C stretching).

Attempt to prepare 2-pyridylmethylidenehydrazono-(4-imidazolyl or 2-methyl-4-imidazolyl)methane (HL^H or HL^{Me})

To a mixture of equivalent amounts of pyridine-2-carboxaldehyde and imidazole-4-carboxaldehyde or 2-methylimidazole-4-carboxaldehyde in 60 mL methanol was added a slightly excess (1.2 equiv.) amount of hydrazine monohydrate. The mixture was refluxed with stirring for 5 h and concentrated under reduced pressure, giving a yellowish residue. The residue was sequentially washed with methanol, acetonitrile, and ethanol and dried over P_2O_5 in a desiccator. ^{1}H NMR analyses of the products from imidazole-4-carboxaldehyde in several attempts gave two spectra (Figure S1 (d)), this further ascertains the fact that, the direct method results in a mixture of compounds. The upper spectrum is likened to the PAA spectrum (Figure S1 (a)), this was confirmed by crystal structures (not reported) from some crystals obtained from the above experiment. The ^{1}H NMR spectra in Figure S1 (d) and (e) confirmed the complicated nature of the products: a mixture of PAA, H_2ICA^H (or H_2ICA^{Me}), and HL^H (or HL^{Me}) which was found to be difficult to separate into the respective azine compounds.

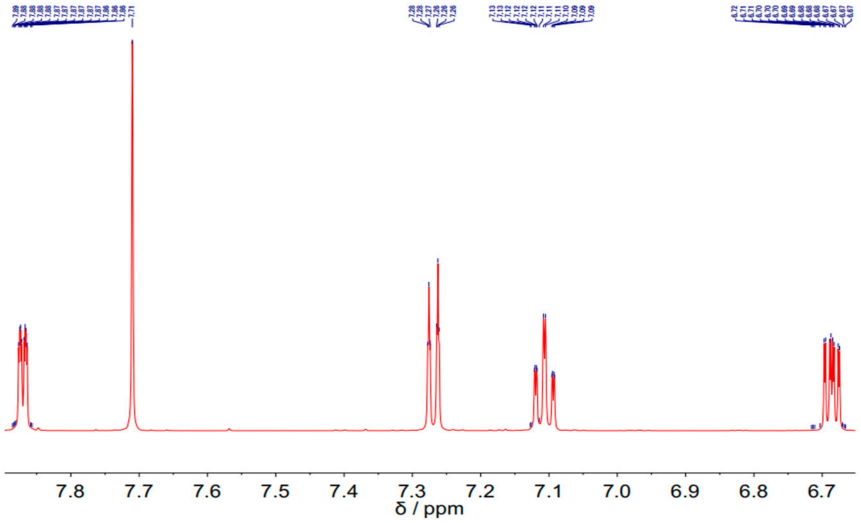

Figure S1. (a) ^{1}H NMR Spectrum of PAA.

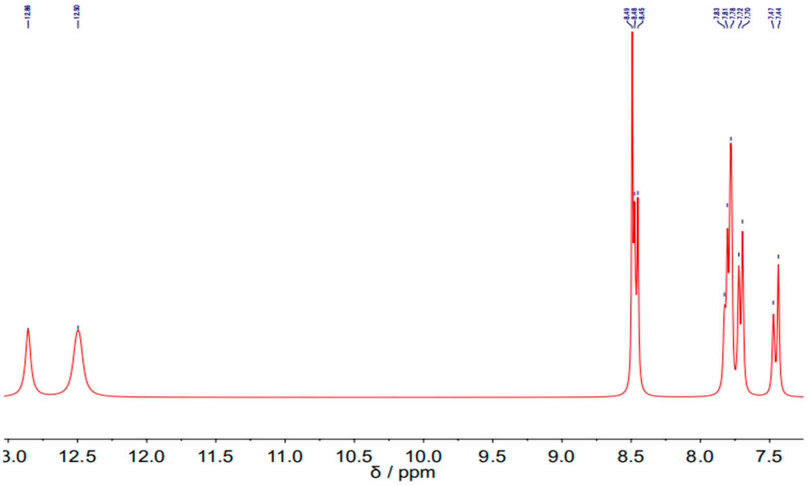

Figure S1. (b) ^{1}H NMR Spectrum of H_2ICA^{H}.

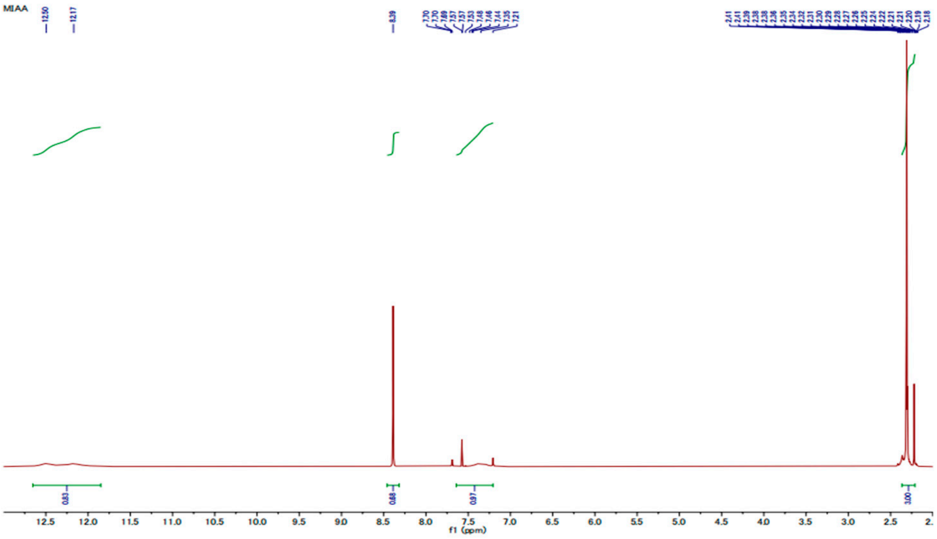

Figure S1. (c) ^{1}H NMR Spectrum of H_2ICA^{Me}.

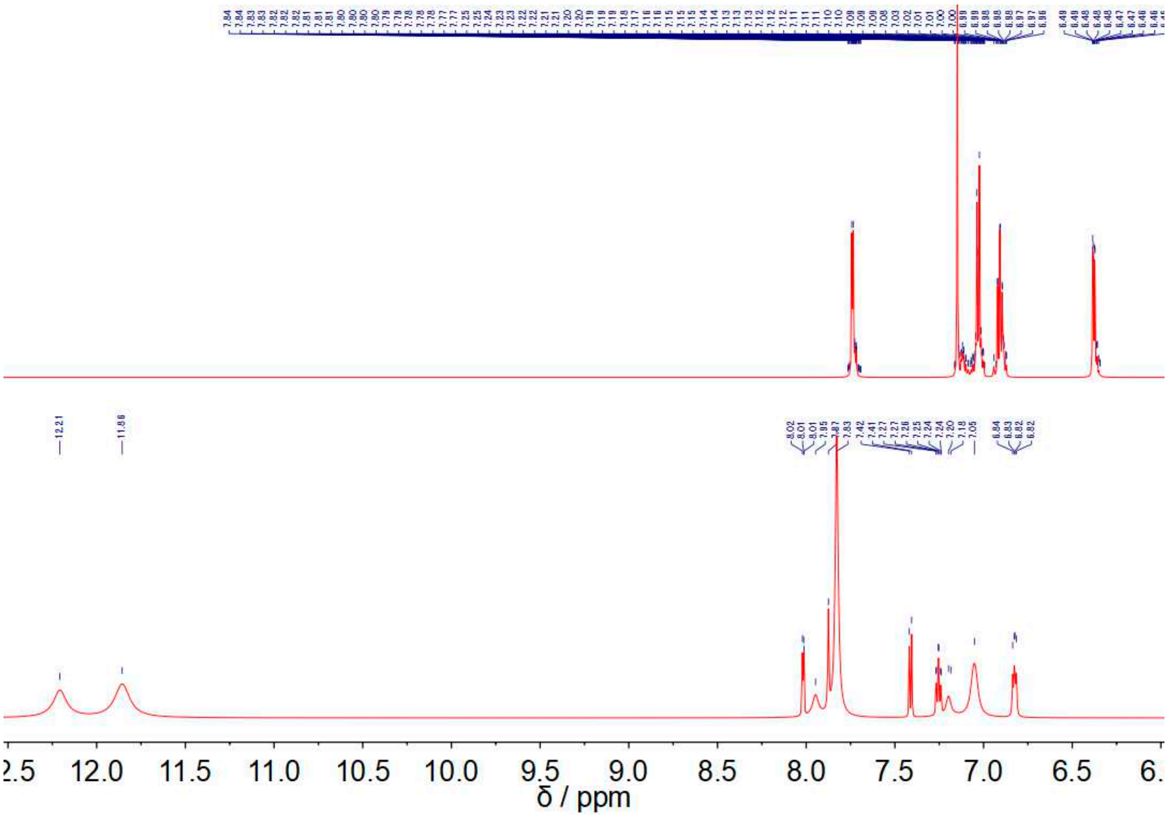

Figure S1. (d) ^{1}H NMR Spectra of direct reaction products (partially separated by fractional crystallization) from pyridine-2-carboxaldehyde, imidazole-4-carboxaldehyde, and hydrazine monohydrate in methanol.

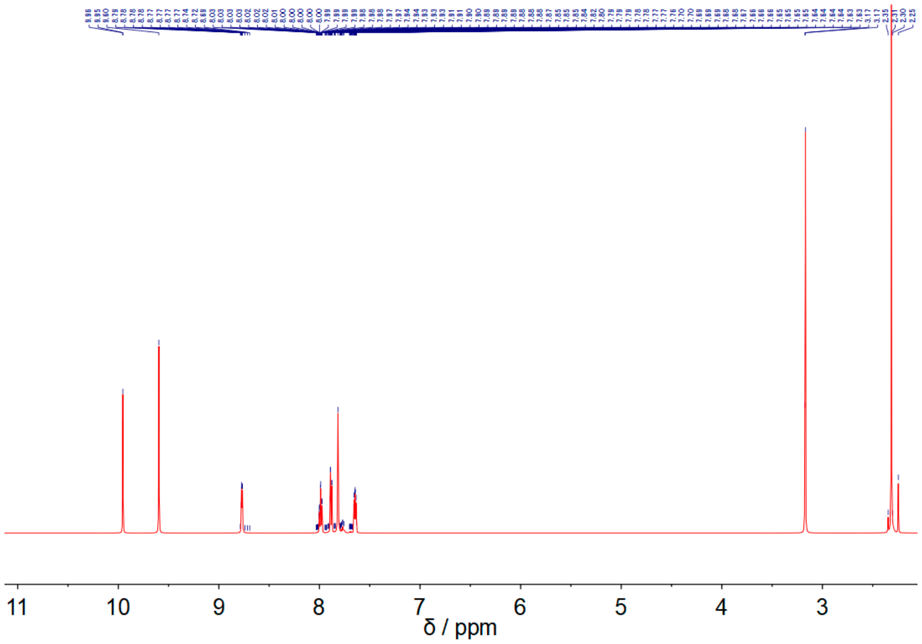

Figure S1. (e) ^{1}H NMR Spectrum of direct reaction products from pyridine-2-carboxaldehyde, 2-methylimidazole-4-carboxaldehyde, and hydrazine monohydrate in methanol.

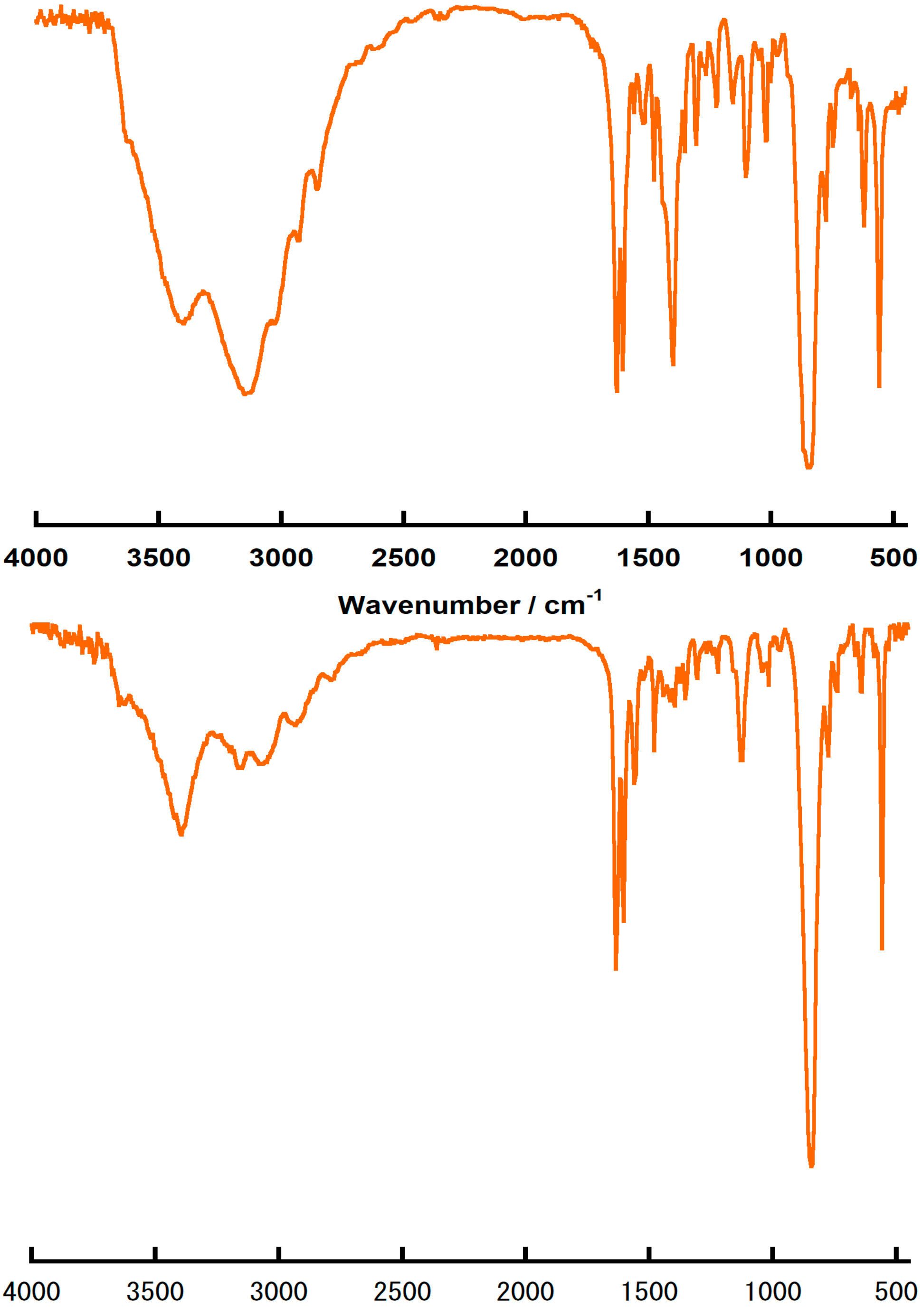

Figure S2. FT-IR spectra of (upper) $[Ni(HL^{H})_2](PF_6)_2$ (**1**) and (lower) $[Ni(HL^{Me})_2](PF_6)_2$ (**2**).

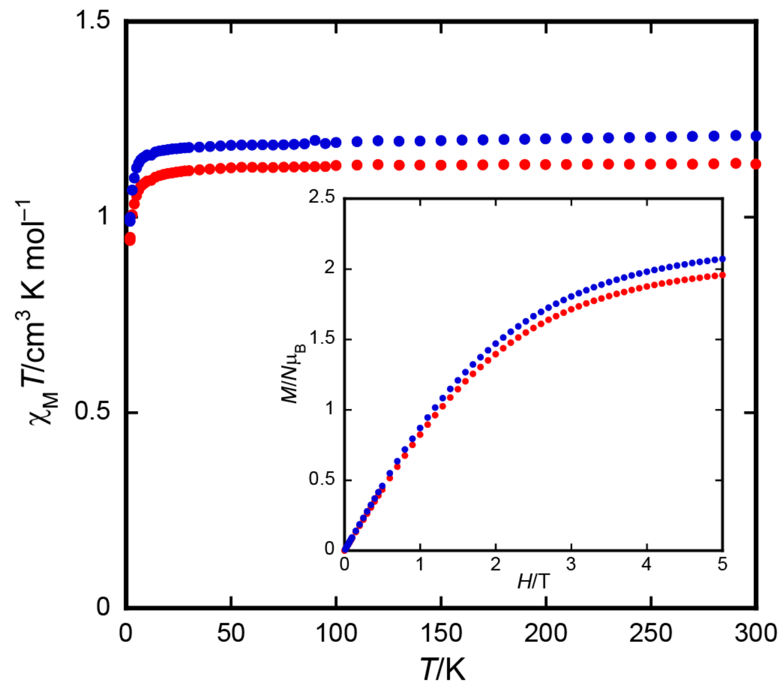

Figure S3. The $\chi_M T$ vs T plots of **1** (red) and **2** (blue) (inset: Magnetizations of **1** (red) and **2** (blue) at 1.9 K).

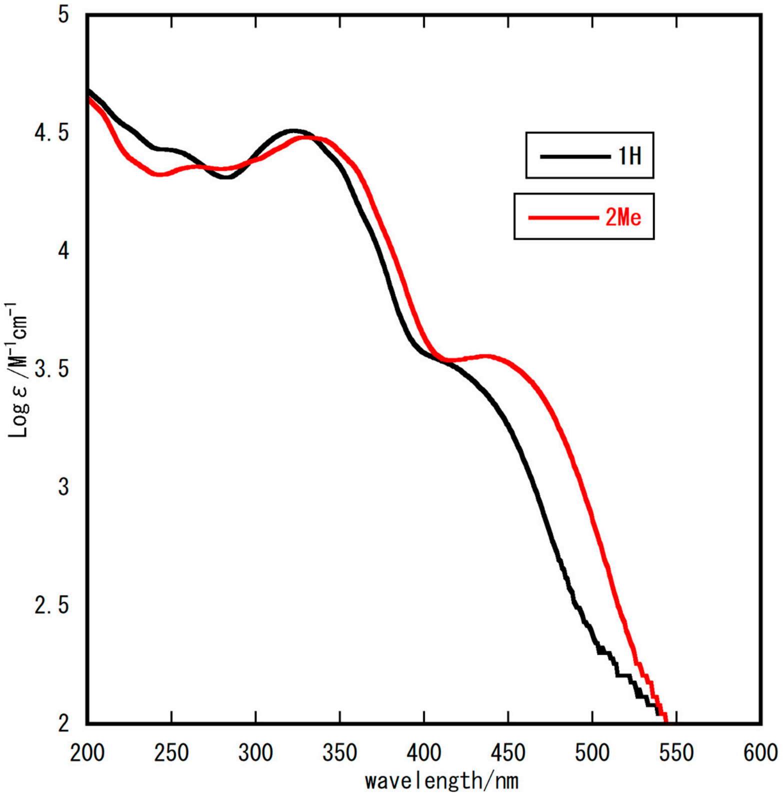

Figure S4. Absorption spectra of **1** (black) and **2** (red) in acetonitrile at room temperature.

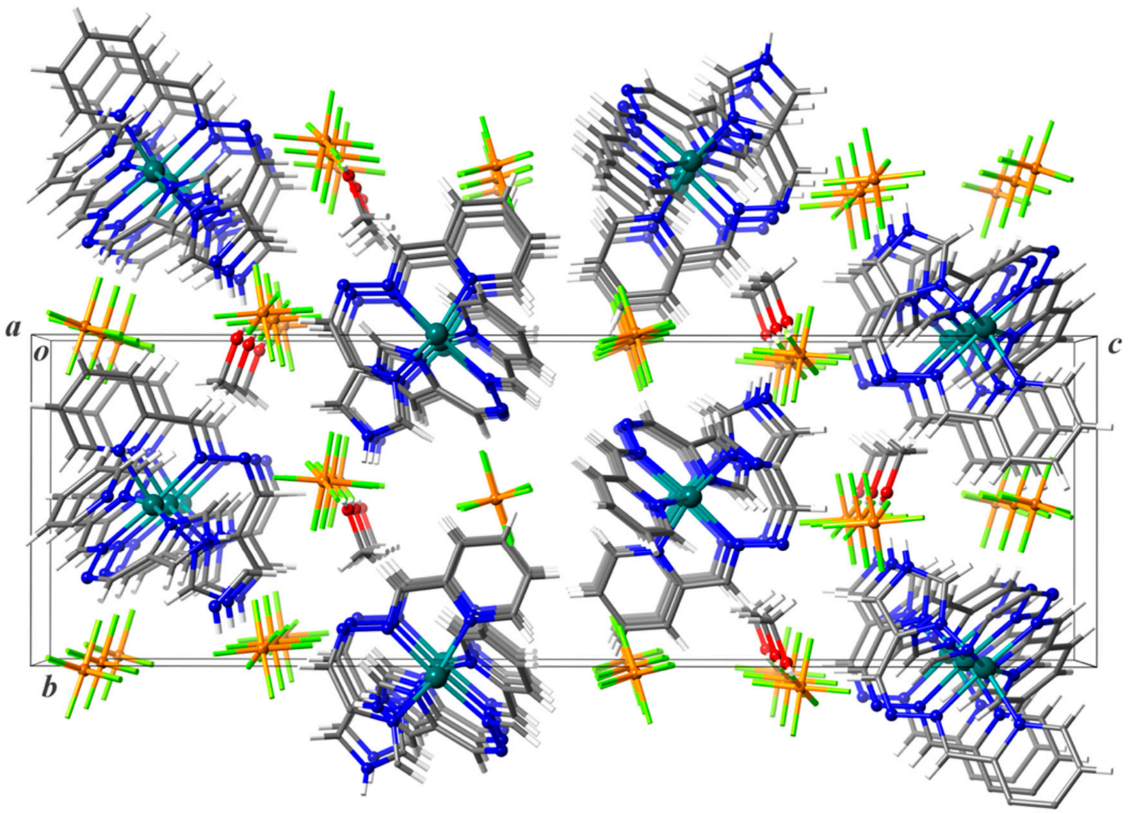

Figure S5. Packing diagram of **1**·MeOH viewed along the crystallographic *a* axis. Color code: Ni, teal; P orange; F, yellow-green; O, red; N, blue; C, gray; H, silver.

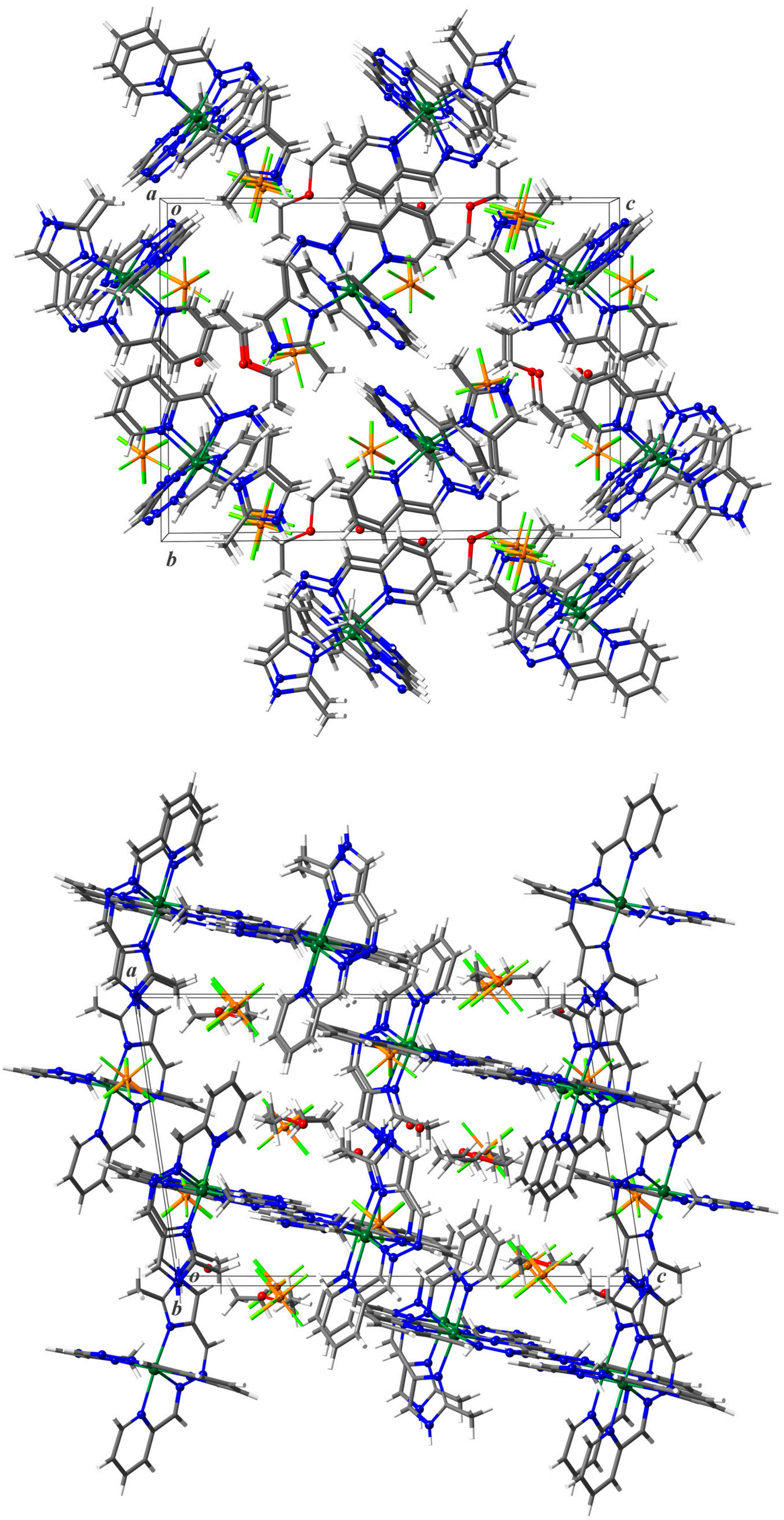

Figure S6. Packing diagrams of **2**·H_2O·Et_2O viewed along (upper) crystallographic *a* axis and (lower) crystallographic *b* axis. Color code: green, Ni; orange, P; yellow-green, F; red, O; blue, N; black, C; silver, H.
